# Intrauterine Exposure to Endocrine-disrupting Chemicals and Risk of Hypospadias: A Pilot Study

**DOI:** 10.1210/jendso/bvaf208

**Published:** 2025-12-12

**Authors:** Cara V Tillotson, Anna Sonnett Fisher, Khue Nguyen, Zoltan Antal, Beizhan Yan, Christina P Carpenter, Patricia Vuguin, Julie Herbstman, Sharon Oberfield

**Affiliations:** Pediatric Endocrinology, Columbia University Medical Center, New York, NY 10032, USA; Pediatric Endocrinology, Columbia University Medical Center, New York, NY 10032, USA; Geochemistry, Columbia University, Lamont-Doherty Earth Observatory, Palisades, NY 10964, USA; Pediatric Endocrinology, Weill-Cornell Medicine, New York, NY 10021, USA; Geochemistry, Columbia University, Lamont-Doherty Earth Observatory, Palisades, NY 10964, USA; Pediatric Urology, Columbia University Irving Medical Center, New York, NY 10032, USA; Pediatric Endocrinology, Columbia University Medical Center, New York, NY 10032, USA; Environmental Health Sciences, Mailman School of Public Health, Columbia University Medical Center, New York, NY 10032, USA; Pediatric Endocrinology, Columbia University Medical Center, New York, NY 10032, USA

**Keywords:** endocrine-disrupting chemicals, neonatology, genitourinary development, hypospadias, bisphenol, paraben

## Abstract

**Context:**

Hypospadias is a common malformation, which can be caused by a disruption of hormone signaling during development. Endocrine disrupting chemicals (EDCs) cross the placenta and can interfere with hormone synthesis and metabolism.

**Objective:**

To evaluate whether intrauterine exposure to environmental phenols and/or parabens is associated with hypospadias.

**Methods:**

This was a case-control pilot study of term infant males with (n = 6) and without (n = 16) hypospadias. Meconium was tested for bisphenol-A (BPA), bisphenol-S (BPS), bisphenol-F (BPF), methylparaben (MePb), and propylparaben (PrPb) using a novel lab procedure.

**Results:**

BPA concentrations were higher in cases vs controls, though this difference was not statistically significant. Higher meconium concentration of BPA was associated with shorter Anogenital distance (AGD); higher BPS and BPA were associated with shorter stretched penile length (SPL). There were no significant differences for BPS, BPF, MePb, or PrPb.

**Conclusion:**

This study demonstrated that EDCs were present in meconium samples, supporting the hypothesis that maternal exposure results in fetal exposure during a time of critical fetal urogenital development. Our data suggests a pattern of higher BPA in cases of hypospadias compared to controls while BPA and BPS were inversely related to AGD and SPL. However, the study is limited by small sample size and therefore was underpowered to detect conclusive differences between the 2 groups. Further studies in EDC exposure and genitourinary differences are warranted.

Hypospadias is among the most common congenital malformations, affecting approximately 1 in 150 live births in the United States, though the etiology of this condition is still largely unknown (1, 2). Approximately 30% of cases are associated with genetic conditions suggesting that nongenetic factors play an important role in the etiology of the majority of hypospadias (3). Genital development in the fetus is guided by an early (week 8-12 gestation), hormone-independent stage and a second stage (week 11-16) during which androgens drive the development of the urethra in males (2). It is during this androgen-driven stage that hypospadias may develop. Animal studies have indicated that hypospadias is caused by a disruption in hormonal signaling during embryonic development (2, 4, 5). Endocrine-disrupting chemicals (EDCs) are exogenous substances that have been shown to interfere with hormone synthesis and metabolism (6-8).

Environmental phenols are a group of chemicals primarily used in the production of plastic products, to which most Americans are exposed in their environment (9-11). Bisphenol-A (BPA) is a widely recognized EDC and has been associated with reproductive and neurodevelopmental birth defects, particularly during in utero exposure in animal model studies and in humans (12-16). Evidence has suggested that 2 BPA alternatives, bisphenol-S (BPS) and bisphenol-F (BPF), may be as or more toxic than BPA (12, 15, 17-20). Due to regulations on BPA in several countries, the use of alternatives such as BPS and BPF in manufacturing has increased in recent years (18). Recent studies have shown an association between intrauterine BPF exposure and increased risk of neurodevelopmental delays, preterm birth, decreased placental weight, and effects on fetal growth parameters (21-23). Prenatal BPS has also been associated with preterm birth and decreased androgen synthesis and reproductive system alterations in animal and human models (19, 24-26). Recent data specifically suggest that intrauterine exposure to bisphenols may be implicated in the development of genitourinary (GU) malformations (12, 27-30). However, there are limited studies evaluating the link between bisphenol exposure and hypospadias development.

Parabens are another group of known EDCs used commonly as a preservative in cosmetic products (31, 32). Previous studies have demonstrated that parabens do cross the placenta as evidenced by their presence in umbilical cord blood, amniotic fluid, placental tissue, and meconium of newborn babies (33-37). Rodent studies have shown paraben exposures were associated with decreased serum testosterone and sperm production as well as histologic changes in reproductive organs (38-40).

In some human studies, prenatal EDC exposure (including parabens and phthalates) has been associated with antiandrogenic effects including decreased anogenital distance (AGD), narrow penile width, increased genital anomalies, and decreased cord blood androgen concentration (testosterone and dehydroepiandrosterone) in male babies (33, 34). However, data is conflicting, and other studies have not shown a significant association between prenatal paraben exposure and shortened AGD (41).

Shortened AGD is a marker for disruption of androgen activity and sexual development and may serve as an indication of male reproductive and sexual health (2, 42, 43). There are several studies linking shortened AGD with decreased fertility, sperm motility, and sperm numbers in adult males (42). These data support parabens as an additional EDC with antiandrogen effects that may be associated with disturbances in the sexual development of the fetus.

Meconium accumulates throughout pregnancy beginning the 11th week of gestation (44). It is a useful matrix for the measurement of exposure to environmental chemicals during the majority of gestation; as such, it may represent a sample of chemicals to which the baby was exposed during development (44).

The objective of this study was to determine whether newborns with hypospadias have higher meconium concentrations of EDCs compared to unaffected controls. A secondary aim of the study was to evaluate AGD and stretched penile length (SPL) in relation to meconium concentrations of EDCs in all participants (cases and controls).

## Methods

### Selection of Participants

This was a multicenter case-control study of full-term infant males. Two New York Presbyterian Hospital sites in New York City were included: Columbia University Medical Center and Weill Cornell Medical Center. Subjects were recruited from July 2022 through December 2023. The study protocol was approved by both sites’ institutional review boards individually prior to initiation of study visits. Cases were recruited upon identification of hypospadias at birth. To account for potential seasonal variation on EDC concentration, recruited control subjects were born within 2 weeks of cases. Inclusion criteria included well term infant males (≥37 weeks gestation) with (cases) or without (controls) hypospadias. Exclusion criteria included prematurity (<37 weeks gestation), known or suspected genetic conditions associated with GU malformations, neonatal intensive care admission, and maternal exposure to medications known or suspected to cause GU malformations based on current available published literature.

### Data Collection

Meconium was collected from the newborn's diaper within the first day of life. All participants used hospital-supplied diapers, which were tested for background levels of bisphenols and parabens via the lab method described later. Meconium was collected using autoclaved metal spoons and placed into glass jars to avoid bisphenol or paraben exposures. Baby wipes were discarded separately without contact with the meconium sample in the diaper to prevent exposure to parabens. The samples were then stored at −80 °C within 20 minutes of collection until time to transport to the lab for analysis.

While not possible to control for all ambient exposure, the meconium collection protocol ensured that samples were as isolated as possible from external EDCs. Sample collection occured by autoclaved metal spoon scraping of meconium, which was directly placed into an autoclaved glass collection vial. This was done to limit any ambient exposure or placement of meconium on any surface aside from the diaper.

A physical exam was performed by trained study staff including 2 practicing pediatric endocrinologists and 1 pediatric endocrine fellow. The exam included evaluating placement of the urethral orifice, classification of hypospadias (if present), determination of the presence of chordee, inspection for other GU malformations, identification of testicular locations, and measurements of SPL and AGD. Birth weight, gestational age, and history of maternal infection were obtained from chart review.

Parents completed a survey that included questions regarding parental exposure to hormones or steroids during pregnancy, medication use in pregnancy, family history of GU malformations, illicit drug use, tobacco or marijuana exposure, race/ethnicity, intention to breastfeed, and parental occupation.

### Lab Analysis

Meconium samples were analyzed for concentrations of BPA, BPS, BPF, methylparaben (MePb), and propylparaben (PrPb). Two hundred fifty mg of homogenized dried meconium was mixed with acetate buffer (pH 5) in a glass tube. The sample was incubated with β-glucuronidase enzyme for 17 hours at 37 °C. After deconjugation, the sample was extracted using QuEChERS (Quick, Easy, Cheap, Effective, Rugged and Safe) (45). QuEchERS extraction included the salting out extraction steps and a follow-up clean-up step to remove the undesired matrix materials like lipids, proteins, and sugars. The samples were effectively purified and ready for liquid chromatography-tandem mass spectrometry (LC-MS/MS) analysis. Ten  mL of acetonitrile was added to the deconjugated mixture prior to adding a pouch of QuEChERS salt package (4 g anhydrous MgSO4 and 1 g NaCL). The mixture was shaken, incubated on ice, and then centrifuged at 3500 rpm for 30 minutes. The supernatant was taken out of the glass tube and added to QuEChERS fatty dispersive-SPE AOAC kit (400 mg PSA, 200 mg C18, and 1200 mg MgSO4). The mixture was shaken to mix and centrifuged for 30 minutes at 3500 rpm and 15 °C. The supernatant sample was then concentrated under N2 stream at 37 °C to dryness. The dry residue was suspended in 200 μL of acetonitrile:water (25:75, v/v) and filtered using filter vials compatible with LC-MS/MS analysis.

LC-MS/MS analysis was performed with the Shimadzu LC system (Shimadzu, Japan) and Qtrap 6500+ (AB Sciex, Framingham, MA, USA) controlled by Analyst software (version 1.6.2, Sciex, Foster City, CA, USA). Chromatography separation was done using Kinetex 1.7 um C18 (50 × 2.1 mm) with a 0.3 mL/min flow rate. The mobile phase A was water and the mobile phase B was methanol:acetonitrile (1:1). The gradient started at 5% B, increased to 100% B in 12 minutes, and was maintained at 100% B for 2 minutes before returning to the initial condition. The system was calibrated for 3 minutes between each run. The mass spectrometer was operated with electrospray ionization in negative mode. Data processing was done on the Multiquant (version 3.0.3, Sciex).

Using this method, we also tested bisphenols and parabens in the top fabric layer of unused diapers. Approximately 100 mg of material was collected from a 2-inch × 2-inch diaper surface. Low concentrations were found in the diaper's top layer, where the meconium sample comes into contact (Table 1). This supports the premise that chemicals found in the diaper did not significantly contribute to concentrations measured in meconium.

**Table 1.** Concentration of EDCs in top layer of unused diapers

Concentration (ng/g)	BPA	BPF	BPS	MePb	PrPb
Diaper top layer 1	4.72	Not detected	1.14	1.36	0.39
Diaper top layer 2	5.10	4.57	0.81	0.80	0.20
Limit of detection (ng/g)	0.09	0.09	0.03	0.03	0.03

Abbreviations: BPA, bisphenol-A; BPS, bisphenol-S; MePb, methylparaben; PrPb, propylparaben.

### Statistical Analysis

Measures of BPA, BPS, BPF, PrPb, and MePb in meconium samples were considered as both continuous variables, where those with values below the limit of detection were imputed (46) with the limit of detection (ie, the lowest value the assay could reliably detect) and categorical variables comparing those with values above the detection limit and below the detection limit. When values were considered as continuous variables, they were natural log transformed to account for their skewed distribution. Welch 2-sample *t*-tests and Fisher's exact tests were run to identify statistical differences between cases and controls when analyzing continuous and categorical data, respectively. To test our main hypotheses, 1-sided *t*-tests were used to determine if having detectable BPA (compared to those with undetectable BPA) was associated with (1) shorter gestational age and (2) shorter AGD. We conducted sensitivity analyses excluding those participants who had no gestational exposure to hormonal medications (n = 4). All statistical analyses were performed in R (version 4.3.3). For all tests, a *P*-value of <.05 was considered statistically significant.

## Results

Twenty-eight subjects were referred to our study, of which 6 cases and 16 controls met inclusion criteria. One subject with hypospadias (case) declined physical exam and questionnaire but consented to meconium testing. There were 3 excluded referrals for each group (cases and controls). Reasons for exclusion included preterm gestation (1), family declined after initial agreement to participate (2), maternal progesterone use in pregnancy (1), and no meconium sample saved for analysis (2).

Subject demographics and questionnaire responses are shown in Table 2. The groups did not differ by race/ethnicity; gestational age; intention to breastfeed; family history of GU malformations; maternal infection status during pregnancy; or maternal use of medications, tobacco, or illicit drugs during pregnancy. Three subjects (1 case and 2 controls) had exposure to levothyroxine in utero. There were no other reported maternal hormones used during pregnancy. Maternal infections included group B streptococcus (GBS) positivity (6 total, all treated perinatally), chorioamnionitis (1), herpes simplex virus (HSV) infection (1), and chlamydial infection (1).

**Table 2.** Characteristics of study participants

Characteristic		Cases (n = 6)	Controls (n = 16)	*P*-value
Gestational age*^a^* (wks)		38.8 (1.47)	38.6(1.20)	.76
Ethnicity/race*^b^*	Hispanic	2 (0.33)	6 (0.38)	1.0
	NH Black	0 (0.00)	2 (0.13)	
	NH White	2 (0.33)	5 (0.31)	
	NH Asian	0 (0.00)	1 (0.06)	
	Other*^c^*	1 (0.17)	2 (0.13)	
	Missing	1 (0.17)	0 (0.00)	
Medications or vitamins used during pregnancy	Yes	3 (0.50)	10 (0.63)	1.0
	No	2 (0.33)	6 (0.38)	
	Missing	1 (0.17)	0 (0.00)	
Maternal infection	Yes	4 (0.67)	9 (0.56)	.61
	No	1 (0.17)	6 (0.38)	
	Missing	1 (0.17)	0 (0.00)	
Breast-fed	Yes	3 (0.50)	15 (0.94)	.13
	No	2 (0.33)	1 (0.06)	
	Missing	1 (0.17)	0 (0.00)	

Abbreviations: NH, non-Hispanic.

^a^Mean (SD).

^b^n (%).

^c^Other includes more than 1 race selected.

### Detection of EDCs in Meconium: Cases vs Controls

EDCs were detected in meconium samples of both cases and controls. Detection status by specific EDC is listed in Table 3. BPA was detected in 50% of cases compared to 27% of controls. There was no difference in detection between groups for BPF or BPS. MePb was detected in 100% of samples (cases and controls), while PrPb was detected in 94% of controls and 67% of cases (*P* = .08). BPA detection was associated with a shorter gestational age (*P* = .04) with mean gestational age of those with detectable BPA being 38 weeks compared to 39 weeks for those with levels below the detection limit.

**Table 3.** Distribution of endocrine-disrupting chemicals by case status

Endocrine-disrupting chemical	Lower limit of detection (ng/g)	Number detected all subjects (%), n = 22	Number detected in cases (%) n = 6	Number detected in controls (%) n = 16	*P*-value cases vs controls
Bisphenol-A (%)	0.09	6 (27)	3 (50)	3 (23)	.28
Bisphenol-S (%)	0.03	7 (32)	2 (33)	5 (31)	1.0
Bisphenol-F (%)	0.09	10 (45)	3 (50)	7 (44)	1.0
Methylparaben (%)	0.03	22 (100)	6 (100)	16 (100)	n/a
Propylparaben (%)	0.03	19 (86)	4 (67)	15 (94)	.08

The mean concentrations of EDCs are presented in Table 4. The geometric mean BPA concentration was 6 times higher in cases (geomean = 3.06 ng/g) compared to controls (0.46 ng/g), though this difference was not statistically significant (*P* = .17). There was no difference in cases and controls in concentrations of BPS, BPF, MePb, or PrPb.

**Table 4.** Meconium EDC concentrations in ng/g for cases and controls

EDC	Concentration (ng/g) cases (hypospadias) n = 6	Concentration (ng/g) controls n = 16	*P*-value cases vs controls
Bisphenol-A	Geomean: 3.07	Geomean: 0.46	.17
	Median: 19.80	Median: 0.09*^a^*	.17
	Range: 0.09*^a^*-322	Range: 0.09*^a^*-793	
Bisphenol-S	Geomean: 0.27	Geomean: 0.13	.31
	Median: 0.03*^a^*	Median: 0.03*^a^*	.34
	Range: 0.03*^a^*-110	Range: 0.03*^a^*-109	
Bisphenol-F	Geomean: 6.05	Geomean: 7.03	.53
	Median: 128.0	Median: 57.6	.50
	Range: 0.09*^a^*-913	Range: 0.09*^a^*-846	
Methylparaben	Geomean: 20.49	Geomean: 16.0	.35
	Median: 27.0	Median: 19.7	.63
	Range: 1.64-90.6	Range: 2.38-145	
Propylparaben	Geomean: 0.59	Geomean: 2.63	.84
	Median: 1.02	Median: 3.64	.84
	Range: 0.03*^a^*-43	Range: 0.03*^a^*-57.5	

Geometric means are presented due to the log-normal distribution of the exposure data.

Abbreviations: EDC, endocrine-disrupting chemical.

^a^Equivalent to limit of detection.

### AGD and SPL in Relation to Concentration of EDCs

**Figure 1.**
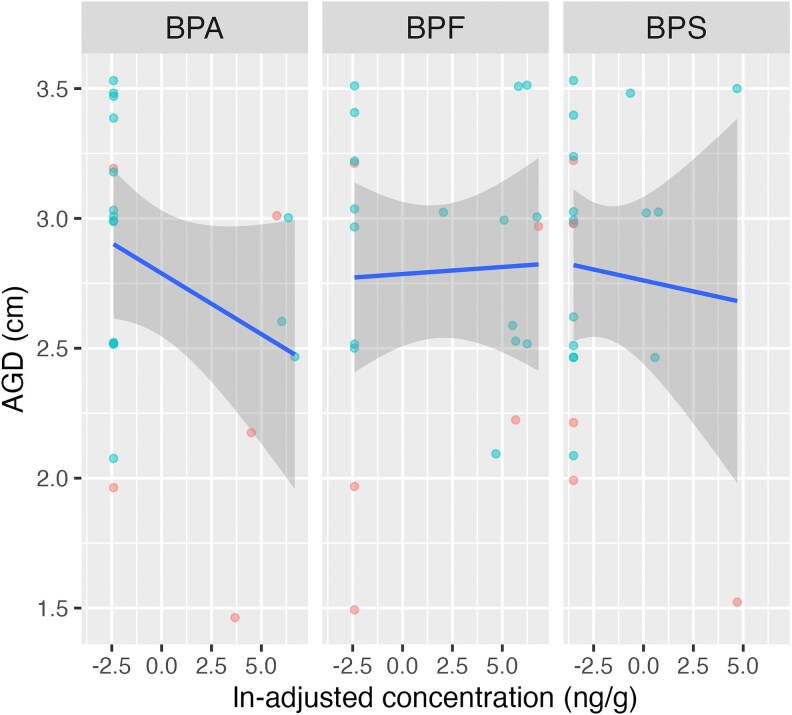
Relationship between anogenital distance in cm and meconium concentration of tested bisphenols. Cases are noted in red and controls in green. Higher meconium levels of BPA and BPS were associated with a shorted anogenital distance. Abbreviations: BPA, bisphenol-A; BPS, bisphenol-S.

**Figure 2.**
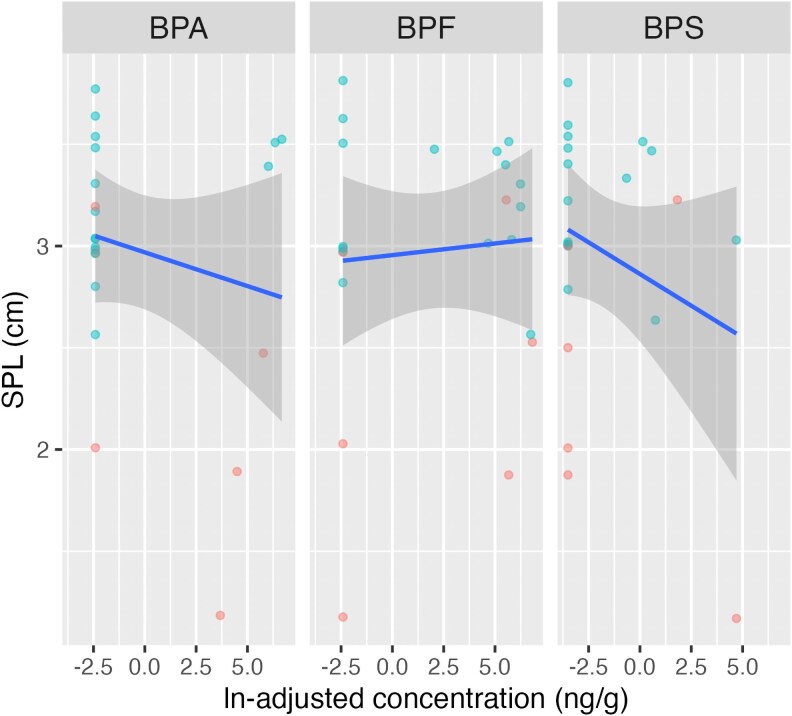
Relationship between SPL in cm and meconium concentration of tested bisphenols. Cases are noted in red and controls in green. Higher meconium levels of BPA and BPS were associated with shorter SPL. Abbreviations: BPA, bisphenol-A; BPS, bisphenol-S; SPL, stretched penile length.

For all subjects (cases and controls), BPA concentration was inversely related to AGD and SPL. BPA and BPS concentrations were both associated with a shorter SPL. These relationships between environmental phenols and AGD and SPL are seen in Fig. 1 and Fig. 2, respectively. Likewise, subjects with detectable levels of BPA had a shorter AGD compared to subjects with below detectable BPA levels; this difference neared statistical significance (2.5 cm vs 3.0 cm, respectively, *P* = .06).

### Sensitivity Analyses

When we restricted to participants who did not have hormonal exposure during gestation, we found similar results, which, in some cases, were slightly stronger than in the full dataset. For example, geometric mean values of BPA was 17.81 ng/g among cases and 0.59 ng/g among controls (*P* = .08) in the subset compared to 3.06 ng/g among cases and 0.46 among controls (*P* = .17). The only notable difference was for BPS, which was not associated with AGD in full dataset but was significantly inversely related (*P* = .05) in the subset that did not have hormonal exposure during gestation.

## Discussion

In this pilot study, we assessed the association between EDC levels measured in meconium and the occurrence of hypospadias and other indicators of endocrine disruption, including AGD and SPL. All EDCs targeted in this study were detectable in some of the meconium samples of newborns, indicating that participants were exposed during the window of susceptibility for the development of hypospadias. These chemicals are frequently found in the environment, and previous studies have demonstrated endocrine-disrupting effects. Our pilot study confirms that maternal exposure does lead to fetal exposure to these chemicals, the effects of which are yet to be fully determined.

Newborns with higher concentrations of BPA were more likely to have hypospadias, though this did not reach statistical significance potentially due to small sample size. Further, AGD and SPL, both markers for androgen exposure, were inversely related to concentrations of BPA. Our findings further support the results of a recent study by Fisher demonstrating that higher maternal BPA levels were associated with shorter AGD and increased risk of cryptorchidism in male offsprings (27). Higher meconium concentration of BPS also had an inverse relationship to SPL, though no similar effect on AGD. Therefore, this alternative to BPA may also have antiandrogen effects on the fetus and should be considered for further studies. Several studies have evaluated AGD and SPL and their relation to various diseases in adults. Shorter AGD has been associated with adult male infertility, while longer AGD in females was associated with higher risk of polycystic ovary syndrome (43). In another study, shorter SPL was noted in adult men with infertility compared to unaffected controls (47). Therefore, bisphenol exposure may be a contributing factor to varying hormonal exposure during fetal development that have lifelong health implications.

This study used a novel matrix (meconium) to study intrauterine exposures to known EDCs. Meconium provides insight into early exposure to these chemicals during critical times in fetal development compared to other means of measurement such as urine or serum, which represents recent exposure due to the short half-life of these chemicals (44) The androgen-driven stage of genital development during which hypospadias and other GU malformations develop (weeks 11-16) aligns with the beginning of meconium accumulation (week 11). Meconium is noninvasive and easy to collect; therefore, meconium is an ideal material for future studies on potential environmental exposures and their relation to fetal development.

Previous studies have focused on maternal exposure to these compounds via sampling the blood, placenta, or urine. Intrauterine BPA has been widely studied and linked to a myriad of complications in the developing fetus including decreased birth weight (48) and neurodevelopmental delays (49, 50). BPF exposure is increasing throughout the world and has been associated with neurodevelopmental delays in infants (21). Both BPS and BPF exposure have been linked to decreased birth weight (18, 22, 23). Remarkably, methylparaben was detected in 100% of the meconium samples in our study. Previous studies demonstrates that increased methylparaben and propylparaben in maternal urine, blood, or the placenta have been associated with increased risk of gestational diabetes and low birth weight males (27, 48, 51). Further, propylparaben has been associated with increased risk of cryptorchidism and reduced AGD in males (27, 30). Our study demonstrates that these substances were all detectable in the meconium of newborn infants, supporting the hypothesis that maternal exposure does indeed reach the fetus and further highlighting the importance of continued research into the consequences of intrauterine exposure. Further studies on the implications of bisphenol and paraben exposure to fetal development are warranted given these known exposure risks. Limitations of this preliminary study include small sample size, which may have contributed to the findings not reaching statistical significance. Likewise, the small sample size increases the potential for detection of random events. There may additionally be yet unknown epigenetic factors associated with the development of hypospadias that contributed to the formation in some patients compared to others when exposed to EDCs. In conclusion, larger studies are needed to explore the potential effects of bisphenols and parabens on GU development.

## Data Availability

Original data generated and analyzed during this study are included in this published article or in the data repositories listed in References.
